# Distributed Phased-Array Radar Mainlobe Interference Suppression and Cooperative Localization Based on CEEMDAN–WOBSS

**DOI:** 10.3390/s25206277

**Published:** 2025-10-10

**Authors:** Xiang Liu, Huafeng He, Ruike Li, Yubin Wu, Xin Zhang, Yongquan You

**Affiliations:** College of Missile Engineering, Rocket Force Engineering University, Xi’an 710025, China; liux_92@163.com (X.L.); 13095315592@163.com (R.L.); binhui369@163.com (Y.W.); zhangxin_shanxi@163.com (X.Z.); youyongquan1997@163.com (Y.Y.)

**Keywords:** distributed radar networks, mainlobe interference suppression, cooperative target localization, blind source separation, low-SNR environments

## Abstract

Mainlobe interference can severely degrade the performance of distributed phased-array radar systems in the presence of strong jamming or low-reflectivity targets. This paper introduces a signal–data dual-domain cooperative antijamming and localization (SDCAL) framework that integrates adaptive complete ensemble empirical mode decomposition with improved blind source separation and wavelet optimization (CEEMDAN-WOBSS) for signal-level denoising and separation. Following source separation, CFAR-based pulse compression is applied for precise range estimation, and multi-node data fusion is then used to achieve three-dimensional target localization. Under low signal-to-noise ratio (SNR) conditions, the adaptive CEEMDAN–WOBSS approach reconstructs the signal covariance matrix to preserve subspace rank, thereby accelerating convergence of the separation matrix. The subsequent pulse compression and CFAR detection steps provide reliable inter-node distance measurements for accurate fusion. The simulation results demonstrate that, compared to conventional blind-source-separation methods, the proposed framework markedly enhances interference suppression, detection probability, and localization accuracy—validating its effectiveness for robust collaborative sensing in challenging jamming scenarios.

## 1. Introduction

With the iterative evolution of new-generation radar technologies, research in the field of electronic countermeasures (ECMs) has demonstrated notably focused hotspots [1,2]. In particular, the application and development of Digital Radio Frequency Memory (DRFM) have rendered active jamming an efficient and widely adopted ECM technique [3]. Depending on the path differences by which electromagnetic interference enters a radar’s antenna pattern, active jamming can be classified into two broad categories: mainlobe jamming and sidelobe jamming [4,5]. While sidelobe jamming countermeasures have matured into well-established technical systems [6,7,8], suppressing mainlobe jamming remains a greater technological challenge. Contemporary monostatic radar platforms predominantly employ countermeasures such as frequency agility [9], feature quantization analysis [10], and polarization-based feature extraction [11] to mitigate mainlobe jamming. However, intrinsic structural limitations—most notably single viewpoints and insufficient information acquisition dimensions—undermine these methods [12]. As jamming techniques continue to evolve in sophistication, the system antijamming efficacy is prone to steep declines under complex ECM scenarios.

To surmount the intrinsic limitations of monostatic radar, distributed radar networking and cooperative antijamming frameworks have gradually emerged at the research frontier. These frameworks can be categorized—according to the level of information fusion—into two principal technical routes: data-level fusion and signal-level fusion. Within the data-level fusion domain, research efforts have predominantly focused on multisource information association and target discrimination algorithms. For example, Ref. [13] first established a false-target recognition model based on spatial distribution differences; Ref. [14] integrated composite azimuth–elevation information from combined active–passive radar systems to construct an interference suppression framework; Ref. [15] proposed a multiple nearest-neighbor–pure-azimuth joint association method for counter-deception jamming; Ref. [16] presented a progressive subset-selection method based on target-localization characteristics, achieving effective false-target discrimination while optimizing radar resource allocation; and Ref. [17] introduced a Frequency-Offset Deception Velocity (FODVB) approach that fuses spatial and frequency information to enhance the discrimination of false targets. Ref. [18] addressed the problem of tracking interference from noncooperative and cooperative false targets within a distributed radar network by proposing a consistency-covariance intersection (CCI) algorithm coupled with a distributed target discrimination scheme.

It is noteworthy that signal-level fusion techniques, by virtue of exploiting richer signal information, exhibit unique antijamming advantages. For instance, Ref. [19] leveraged distinctions between true targets and deceptive jammers in the Doppler-velocity domain to develop a joint target-authentication model for active–passive radar systems; Ref. [20] employed convolutional neural networks to extract multidomain features from radar echoes, enabling multistatic radar resilience against deception jamming; Ref. [21] proposed a noise-subspace projection-based interference suppression method that effectively discriminates false targets according to differences in spatial scattering characteristics; Ref. [22] utilized the complex envelopes of any two targets across different receivers for correlation testing to identify false targets; Ref. [23] applied a differencing principle to estimate amplitude sequences of received signals at different radar nodes and constructed test statistics to detect deception jamming under a given false-alarm probability; Ref. [24] fused multidomain features of echo signals via a residual convolutional neural network augmented with an attention mechanism, achieving precise identification of multiple simultaneous mainlobe deception jammers; and Ref. [25] combined the Neyman–Pearson criterion with a hybrid NP-MAP criterion and utilized polarization optimization to accurately identify and suppress active deception jamming in high-JNR environments.

Despite these advances, most existing antijamming methods are predicated on the theoretical assumption of an ideal received signal-to-noise ratio (SNR). In practical electromagnetic environments, a radar receiver must contend with the coupling effects of multiple interference sources—including strong-power active jamming, adjacent-frequency/co-frequency signal crosstalk, industrial electromagnetic noise, and complex ground/sea clutter—resulting in the effective signal often being submerged beneath a noise baseline (typical SNR < 3 dB). Under such low-SNR conditions, traditional antijamming systems exhibit the following technical limitations: (1) significant degradation of target-localization accuracy due to signal distortion; (2) sharp declines in weak-target detection probability; and (3) rapid attenuation of mainlobe jamming suppression efficacy.

In response to the aforementioned challenges, this paper proposes a signal–data dual-domain cooperative antijamming framework for phased-array (PA) radar, comprising two core modules: (1) at the interference suppression level, a joint processing method based on complete ensemble empirical mode decomposition with adaptive noise combined with wavelet-optimized blind source separation (CEEMDAN–WOBSS) is developed to achieve strong jamming suppression under low-SNR conditions via noise-baseline reconstruction and signal-subspace decomposition, and (2) at the cooperative localization level, a cooperative positioning framework is devised for bistatic and multistatic radar configurations, leveraging measurement data from distributed radar-network nodes to realize three-dimensional target localization.

Blind source separation (BSS) techniques, first proposed in the 1980s, have since evolved into a mature theoretical framework in fields such as communications signal processing [26]. Their core mechanism relies on the assumption of statistical independence to recover latent source signals from mixed observations. In the radar antijamming domain, Ref. [27] first estimated the number of signal sources via the Gerschgorin radius method to satisfy BSS conditions, then applied the JADE algorithm—based on fourth-order cumulant-matrix approximate joint diagonalization—to separate radar-received signals. Ref. [28] constructed a mixed polarization–signal model by exploiting the polarization differences between interference signals and target echoes, achieving antijamming through interference reconstruction followed by BSS. Ref. [29] proposed a jointly improved BSS technology based on transmit–receive coordination, employing a dynamic particle swarm optimization algorithm to derive the optimal extraction vector for target echoes, thereby effectively suppressing multiple false-target deception jammers. To address direction-of-arrival (DOA) estimation challenges under mainlobe jamming, Ref. [30] introduced a method based on spatiotemporal multichannel BSS, jointly optimizing signal separation and phase-statistical features to obtain both range and DOA information. Ref. [31] combined sum-and-difference beamforming with subarray blind source separation and monopulse techniques to counter high-power mainlobe jamming, enabling DOA estimation of weak targets. Ref. [32] proposed a JADE-BSS and energy-detection joint approach to separate target and jamming signals in sum-and-difference beams, and subsequently extracted uncontaminated signal segments for monopulse radar DOA estimation.

All the above references pertain to monostatic radar mainlobe jamming suppression based on BSS algorithms. For distributed, multistatic radar-network systems, Ref. [33] constructed a specialized subarray structure for radar-received signals, applied BSS to separate interference and target signals, and then employed sparse signal reconstruction to jointly estimate target DOA. Ref. [34] first compensated for the relative delays of each radar’s interference signals using correlation methods, then applied JADE-BSS to separate target and interference signals. Ref. [35] proposed a Maximum Signal-to-Noise Ratio BSS (MSNR-BSS) method in combination with CFAR detection to achieve signal-level antijamming and performed spatial localization via radial distance estimation. Gao et al. [36] extended the MSNR-BSS algorithm to frequency-diverse array multiple-input-multiple-output (FDA-MIMO) radar for effective signal separation.

In summary, BSS methods have been widely applied to radar signal processing; however, prevailing mainstream algorithms still face two major technical bottlenecks. First, the JADE algorithm—based on fourth-order cumulant–matrix joint diagonalization—requires constructing a cumulant matrix of dimension $O(N^{4})$ (where *N* is the number of channels), resulting in high computational complexity. Second, BSS algorithms exhibit strong sensitivity to the SNR: empirical studies show that when the SNR<5dB, the signal separation error rate increases drastically, severely degrading interference suppression performance. More importantly, within the domain of distributed radar-network cooperative antijamming, each node’s radar is more likely to receive echoes under low-SNR conditions, yet only a limited number of publications address distributed radar antijamming and cooperative localization specifically under such conditions. To address this challenge, this study examines distributed phased-array (PA) radar performance under severe interference and low-SNR detection scenarios (typical SNR∈[−5dB,5dB]). We propose a signal–data dual-domain cooperative antijamming and localization (SDCAL) framework with the following innovations:

(1) To address the failure of traditional empirical mode decomposition (EMD) due to the nonstationary nature of PA radar-received signals, we propose a noise-adaptive complete ensemble empirical mode decomposition method combined with an improved wavelet-based signal preprocessing approach, thereby enabling modal decomposition for PA radar echoes.
(2) For low-SNR scenarios, we introduce a CEEMDAN combined with wavelet-optimized maximum-SNR blind source separation (CEEMDAN–WOBSS) joint processing method, which enhances the separation performance of PA radar-received signals and strengthens interference suppression capabilities under low SNRs.
(3) In order to address the cooperative localization problem in distributed radar networks under low-SNR conditions, a signal–data dual-domain cooperative antijamming and localization (SDCAL) framework is constructed to integrate signal-level interference suppression with data-level collaborative positioning, thereby achieving accurate target spatial localization.

The subsequent sections of this paper are structured as follows. Section 2 presents the received-signal model for the distributed radar system. Section 3 describes the SDCAL processing flow and provides a detailed derivation of the signal-domain antijamming algorithm. Section 4 outlines the target detection approach and establishes the data-domain cooperative localization framework under low-SNR conditions. Section 5 presents detailed numerical simulation results to validate the effectiveness of the proposed method. Finally, Section 6 summarizes the conclusions of this work.

Current research is limited to the academic field of radar signal processing for civilian sensing applications—such as environmental monitoring and disaster response—which offers clear societal benefits and does not pose a threat to public health or national security. The authors acknowledge the dual-use potential of the advanced signal-separation and localization algorithms presented here and confirm that all necessary precautions have been taken to prevent potential misuse. In accordance with relevant national and international DURC regulations, the authors advocate for responsible deployment, ethical considerations, regulatory compliance, and transparent reporting to mitigate misuse risks and foster beneficial outcomes.

## 2. Radar Signal Model

The radar network studied in this paper is a distributed phased-array system featuring multiple cooperative transmitters and receivers. Specifically, the network comprises *m* phased-array (PA) nodes, each operating as a transceiver capable of transmitting and receiving waveforms. It is assumed that all nodes achieve time synchronization through pre-coordination or network synchronization and share common waveform parameters.

**Figure 1:**
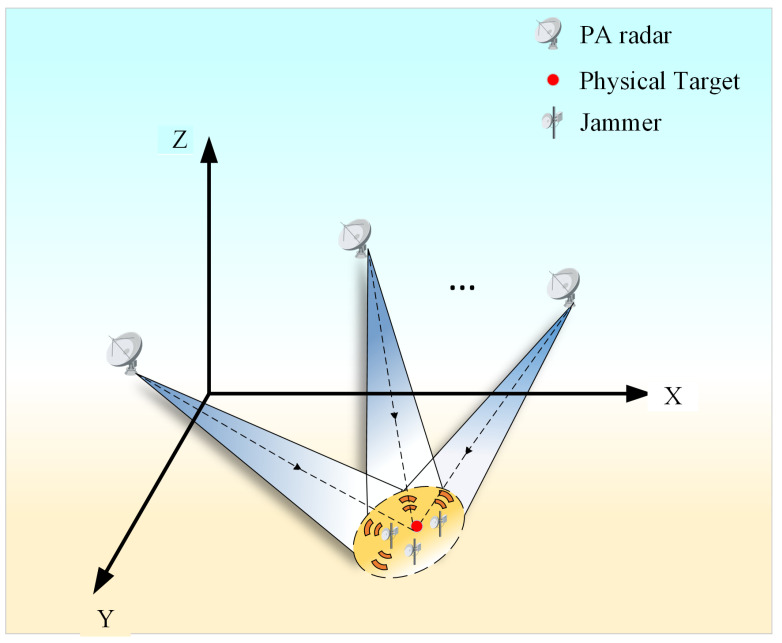
Schematic diagram of distributed radar detection.

As shown in Figure 1, the distributed multistatic radar system comprises *m* PA radars, *K* jammers, and *P* targets. Each PA radar’s antenna aperture consists of *S* uniformly spaced linear array elements. It is assumed that both targets and jammers lie in the far-field region, with the jammers distributed around the targets. The position of the *n*th PA radar is denoted by $\left(x_{n}^{r},y_{n}^{r},z_{n}^{r}\right)$, the *k*th jammer by $\left(x_{k}^{j},y_{k}^{j},z_{k}^{j}\right)$, and the *s*th target by $\left(x_{s}^{t},y_{s}^{t},z_{s}^{t}\right)$.

Assume that the transmitted waveform from each PA radar is $S_{m}\left(t\right)$. Since targets are located in the far-field, the path length from a given target to all array elements of a PA radar can be approximated as equal. Therefore, the target-echo signal received at the *s*th element of the *m*th radar is expressed as

(1) $$r_{m,s}=\sum_{p=1}^{P}\alpha_{m,s,p}s_{p}^{t}\left(t-\tau_{m,p}\right)\exp\left\{-j2\pi \frac{(l-1)d}{\lambda }\sin\theta_{m,p}^{t}\right\}$$

where $s_{p}^{t}\left(t\right)$ denotes the PA radar’s transmit signal, $\alpha_{m,s,p}$ is the complex amplitude of the received target echo, $\tau_{m,p}$ is the time delay of the echo at the *m*th radar, λ is the signal wavelength, *d* is the inter-element spacing, and $\theta_{m,p}^{t}$ is the direction-of-arrival (DOA) of the *p*th target echo at the *m*th radar.

Jammers are assumed to lie in the same far-field region and to have negligible radar cross-sections (RCSs). Consequently, the radar will receive both the target echo and the interference transmitted by the *k*th jammer. The interference signal received at the *s*th element of the *m*th radar is given by

(2) $$J_{m,s}=\sum_{k=1}^{K}\beta_{m,s,k}s_{k}^{j}\left(t-\tau_{m,k}\right)\exp\left\{-j2\pi \frac{(l-1)d}{\lambda }\sin\theta_{m,k}^{j}\right\}$$

where $s_{k}^{j}\left(t\right)$ denotes the *k*th jammer’s transmit waveform, $\beta_{m,s,k}$ is the complex amplitude of the received interference, $\tau_{m,k}$ is the interference time delay at the *m*th radar, and $\theta_{m,k}^{j}$ is the DOA of the *k*th mainlobe jammer at the *m*th radar.

Let the received-signal vector at the *m*th PA radar be

(3) $$X_{m}={\left[x_{m,1}\left(t\right),x_{m,2}\left(t\right),\cdots ,x_{m,s}\left(t\right)\right]}^{T}$$

where $x_{m,s}\left(t\right)$ represents the total signal (target echo plus interference) received at the *s*th element. Then $X_{m}$ can be written in compact form as

(4) $$X_{m}=A_{m}S_{m}+n_{m}=A_{R}S_{R}+A_{J}S_{J}+n_{m}$$

where $A_{m}$ denotes the S×(L+K) complex mixing matrix for the *m*th radar, $S_{m}$ is the (L+K)-dimensional vector of source signals (all target echoes and jammer waveforms) impinging on the *S* elements, and $n_{m}$ is a complex Gaussian white-noise vector with zero mean and covariance $\sigma_{m}^{2}$.

(5) $$A_{m}=\left[A_{R},A_{J}\right]$$

(6) $$A_{R}=\left[\alpha_{m,s,1}\varphi^{t}\left(\theta_{m,1}\right),\alpha_{m,s,2}\varphi^{t}\left(\theta_{m,2}\right),\ldots ,\alpha_{m,s,P}\varphi^{t}\left(\theta_{m,P}\right)\right]$$

(7) $$A_{J}=\left[\beta_{m,s,1}\varphi^{j}\left(\theta_{m,1}\right),\beta_{m,s,2}\varphi^{j}\left(\theta_{m,2}\right),\ldots ,\beta_{m,s,K}\varphi^{j}\left(\theta_{m,K}\right)\right]$$

(8) $$\varphi^{t}\left(\theta_{m,p}\right)=\exp\left\{j2\pi \frac{(l-1)d}{\lambda }\sin\theta_{m,p}^{t}\right\}$$

(9) $$\varphi^{j}\left(\theta_{m,k}\right)=\exp\left\{j2\pi \frac{(l-1)d}{\lambda }\sin\theta_{m,k}^{j}\right\}$$

(10) $$S_{m}={\left[S_{R}\left(t\right),S_{J}\left(t\right)\right]}^{T}=\left[\begin{array}{c} s_{1}^{r}\left(t-\tau_{n,1}\right)\\ s_{2}^{r}\left(t-\tau_{n,2}\right)\\ \vdots \\ s_{P}^{r}\left(t-\tau_{n,P}\right)\\ s_{1}^{j}\left(t-\tau_{n,1}\right)\\ s_{2}^{j}\left(t-\tau_{n,2}\right)\\ \vdots \\ s_{k}^{j}\left(t-\tau_{n,K}\right) \end{array}\right]$$

(11) $$n_{m}=\left[\begin{array}{c} n_{m,1}\left(t\right)\\ n_{m,2}\left(t\right)\\ \vdots \\ n_{m,S} \end{array}\right]$$

where ${\left(\;\cdot \;\right)}^{T}$ is the transpose operation.

## 3. Signal-Domain Antijamming for Distributed Radar

### 3.1. Algorithm Flow

**Figure 2:**
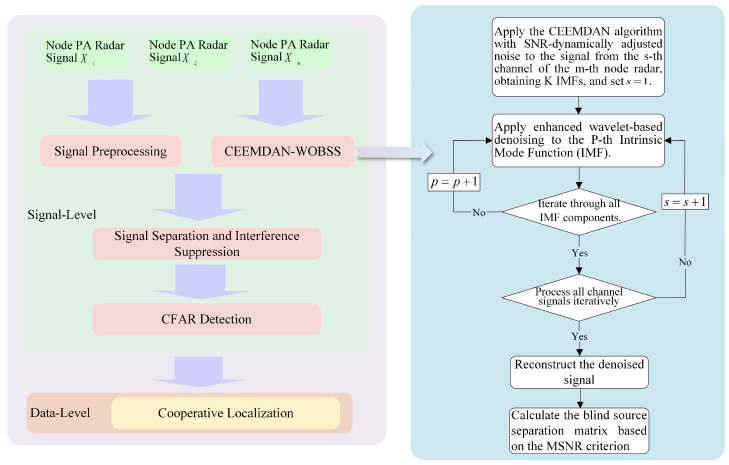
Signal–data cooperative antijamming and localization framework (SDCALF).

To address interference suppression and cooperative localization in distributed radar networks under low-SNR conditions, the SDCAL framework is proposed, as illustrated in Figure 2. This framework consists of two stages: signal-level interference suppression at each networked radar node and data-level cooperative localization across the distributed radar network. The algorithm process is as follows:

1. Perform mean-subtraction processing on the PA radar reception signals from each node in the distributed radar system to eliminate DC components, followed by pre-whitening processing to decorrelate the components of the reception signals.
2. Apply joint CEEMDAN-WOBSS processing to the echo signals received by the PA radar, then calculate the separation matrix based on the maximum signal-to-noise ratio criterion and perform blind source separation on the whitened signals.
3. CFAR detection is performed on all channel signals after pulse compression, and the detected signal channels are superimposed to obtain the radial distance of the target. Finally, the spatial position of the target is jointly calculated using the radial distances of the target from two or three PA radars in the distributed radar network.

### 3.2. PA Radar-Received Signal Preprocessing

BSS requires the mixing matrix $A_{m}$ to be of full column rank. Equivalently, the number of receive channels *S* must exceed the total number of source signals, and at most, one source may be Gaussian, while all source signals must be statistically independent. In the distributed multistatic radar system considered here, each single-site PA radar has *S* receive elements, the detection region contains *K* jammers and *P* targets, and it is assumed that S>P+K. Since mainlobe suppression jamming is modeled as noise-modulated interference, the interference and target echo signals are statistically independent; the Gaussian white noise component serves as the unique Gaussian source.

Let each PA radar have *S* receive antennas. Suppose there are *P* real targets and *K* jammers in the detection region, with S>P+K. The received signal at the *m*th PA radar can then be written in matrix form as

(12) $$X_{m}=A_{m}S_{m}^{o}+n_{m}$$

where

- $X_{m}$ is the S×D received-signal matrix, $X_{m}=\left[x_{1}^{m}\left(t\right),x_{2}^{m}\left(t\right),\cdots ,x_{s}^{m}\left(t\right)\right]$, and *D* denotes the number of fast-time samples;
- $S_{m}^{o}$ is the (P+K)×D source-signal matrix containing all target-echo and jammer signals impinging on the *S* array elements;
- $A_{m}$ is the S×(P+K) mixing matrix for the *m*th radar;
- $n_{m}$ is the S×D complex Gaussian white-noise matrix with zero mean and covariance $\sigma_{m}^{2}$.

Prior to blind source separation, the received signals in each element channel of the PA radar undergo zero-mean centering (de-meaning) and whitening. De-meaning (centering) removes any DC component from $X_{m}$.

(13) $${\bar{X}}_{m}=X_{m}-E\left[X_{m}\right]$$

where E[·] denotes the expectation operator. Whitening (pre-whitening) then decorrelates the zero-mean data. Let $C_{XX}$ be the covariance matrix of ${\bar{X}}_{m}$. Denote by U the S×n matrix whose columns are the *n* largest eigenvectors of $C_{XX}$, and let $\Lambda =\mathrm{diag}(d_{1},d_{2},\cdots ,d_{n})$ be the corresponding diagonal matrix of eigenvalues. The whitening matrix Q is then given by

(14) $$Q=\Lambda^{-1/2}U^{H}$$

and the whitened observation matrix is

(15) $$X_{m}^{\prime }=Q{\bar{X}}_{m}$$

Once the preprocessed observation $X_{m}^{\prime }$ is obtained, the separation matrix W is computed according to a chosen BSS algorithm, and the target echo is extracted by applying W to the observations of the original PA radar’s individual antenna element channel signals. In the MSNR-BSS algorithm, the SNR criterion is formulated as an error-minimization cost function:

(16) $$J\left(w\right)=-\frac{E\left[|w^{T}{x|}^{2}\right]}{E\left[|w^{T}{n|}^{2}\right]}$$

where W is a separation vector, x is the mixed observation, and n is the noise component. It can be shown that the convergence of W depends directly on the whitened signal’s SNR level. Moreover, the whitening matrix Q depends on the second-order statistics of the received signal:

(17) $$C_{XX}=E\left[{X^{\prime }}_{m}{X^{\prime }}_{m}^{H}\right]=AC_{s}A^{H}+\sigma^{2}I,$$

where $C_{s}=E\left[ss^{H}\right]$ is the source-signal covariance matrix, A the mixing matrix, $A^{H}$ is its Hermitian transpose, $\sigma^{2}$ is the noise power, and I is the identity matrix.

Under low-SNR conditions—i.e., $\sigma^{2}\gg ∥AC_{s}A^{H}∥$—the whitening process attenuates the target-signal subspace in $X_{m}^{\prime }$, causing W to converge to a suboptimal solution and preventing effective separation of signal and interference subspaces. To address this, the proposed CEEMDAN–WOBSS method attenuates the noise-baseline power in the whitened observations and reconstructs the rank characteristics of the signal covariance matrix, thereby enabling accurate discrimination and extraction of both target and interference components.

### 3.3. Signal Separation and Interference Suppression

Each received signal sample $x^{\left(s\right)}\left(t\right)$ at the *s*th array element of every PA radar node undergoes CEEMDAN-based decomposition with iterative added noise. Denote by $w^{i}\left(t\right)$ the white-noise sequence added at the *i*th iteration, whose standard deviation is adaptively chosen to achieve a target SNR level $\beta_{i}$. The noisy observation sequence is formed as

(18) $$x_{k}^{\left(s\right)}\left(t\right)=x^{\left(s\right)}\left(t\right)+\beta_{i}D_{k}\left[w^{i}\left(t\right)\right]$$

where $D_{k}\left(\;\cdot \;\right)$ extracts the *k*th intrinsic mode function (IMF) via an M-order EMD. The dynamic coefficient $\beta_{i}$ is computed from the ratio of signal-to-noise energy in the residual:

(19) $$\beta_{i}=\left\{\begin{array}{cc} \varepsilon \;\cdot \;\frac{x^{\left(s\right)}\left(t\right)x^{\left(s\right)}{\left(t\right)}^{T}}{\left(w^{\left(i\right)}\right){\left(w^{\left(i\right)}\right)}^{T}}, & \mathrm{if}\;i=0\\ \varepsilon \;\cdot \;\frac{R_{i}\left(t\right)R_{i}{\left(t\right)}^{H}}{\left(w^{\left(i\right)}\right){\left(w^{\left(i\right)}\right)}^{H}}, & \mathrm{if}\;i\geq 1 \end{array}\right.$$

where $R_{i}\left(t\right)$ is the residual after the (i−1)th decomposition, ε is the initial noise amplitude, ε is typically set to 20% of the standard deviation of the received signal to ensure that noise injection effectively aids modal decomposition without excessively contaminating the original signal characteristics.

After forming M noisy realizations, a first-order decomposition yields M IMF estimates. Their ensemble average defines the first-order IMF of the original signal:

(20) $${\bar{\mathrm{IMF}}}_{1}^{\left(s\right)}=\frac{1}{M}\sum_{k=1}^{M}D_{k}\left[x^{\left(s\right)}\left(t\right)+\beta_{i}D_{k}\left[w^{\left(i\right)}\left(t\right)\right]\right]$$

The first residual is then

(21) $$R^{\left(1\right)}\left(t\right)=x^{\left(s\right)}\left(t\right)-{\bar{\mathrm{IMF}}}_{1}^{\left(s\right)}\left(t\right)$$

By injecting new noise into $R^{\left(1\right)}$ and repeating the above steps, all IMF orders can be extracted. A time-varying noise power strategy is implemented using the dynamic parameter $\beta_{i}$—maintaining a high noise level in the early (high-frequency) stages and gradually attenuating it in the later (low-frequency) stages—to suppress mode aliasing and minimize residual noise in the final reconstruction. Previous studies have confirmed that each IMF retains complete echo signal information, so all IMFs are retained to achieve accurate signal reconstruction.

In order to enhance BSS performance under low-SNR conditions, a multi-element parallel processing scheme is adopted. Each IMF is processed via improved wavelet-threshold denoising:

(22) $${\hat{w}}_{j,k}=\left\{\begin{array}{cc} \mathrm{sgn}\left(w_{j,k}\right)\left(|w_{j,k}|-\frac{\lambda }{\alpha {\left(w_{j,k}-\lambda \right)}^{2}/\lambda +1}\right), & \mathrm{if}\;|w_{j,k}|\;\geq \lambda \\ 0, & \mathrm{if}\;|w_{j,k}|\;<\lambda \end{array}\right.$$

where α>1 balances denoising strength against signal fidelity. The threshold λ was set according to Donoho’s universal-threshold rule, i.e.,

$$\lambda =\sigma \sqrt{2\ln(N)},$$

where σ denotes the estimated noise standard deviation and *N* is the signal length. This threshold is continuous over its domain and reduces to identity for large amplitudes, thus avoiding the constant-bias drawback of soft-thresholding while preserving oscillation suppression.

All denoised IMFs are then recombined:

(23) $${\hat{x}}^{\left(s\right)}\left(t\right)=\sum_{k=1}^{M}R\left({\mathrm{IMF}}_{k}^{\left(s\right)}\right)$$

where R(·) denotes the inverse wavelet reconstruction operator. After zero-mean centering and whitening, the matrix of preprocessed signals ${\hat{X}}^{\prime }$ forms the input to MSNR-BSS. Define

(24) $$\mathrm{SNR}=10\log\left(\frac{W{\hat{X}}^{\prime }\;\cdot \;{\left({\hat{X}}^{\prime }\right)}^{H}W^{T}}{W\left({\hat{X}}^{\prime }-X^{\prime }\right)\;\cdot \;{\left({\hat{X}}^{\prime }-X^{\prime }\right)}^{H}W^{T}}\right)$$

where $C={\hat{X}}^{\prime }\;\cdot \;{\left({\hat{X}}^{\prime }\right)}^{H}$, $\;\hat{C}=\left({\hat{X}}^{\prime }-X^{\prime }\right)\;\cdot \;{\left({\hat{X}}^{\prime }-X^{\prime }\right)}^{H}$,$\;V=WCW^{T}$ and $\;U=W\hat{C}W^{T}$. Setting $\frac{\partial F}{\partial W}=0$ yields the eigenvalue problem

(25) $$WC=\frac{V}{U}WC$$

The eigenvectors of $\hat{C}\;\cdot \;C^{-1}$ (rows of W) will separate the sources [37]. The estimated source matrix is

(26) $$Y=WX^{\prime }$$

and each channel $y_{m,l}\left(t\right)$ is pulse-compressed against the transmitted waveform $s_{l}^{r}\left(t\right)$:

(27) $$g_{m,l}\left(t\right)=\int y_{m,l}\left(\tau \right)s_{l}^{r}\left(t-\tau \right)\;d\tau$$

further suppressing residual interference and yielding high-fidelity target echoes.

## 4. Target Detection and Cooperative Localization

Following signal separation and pulse compression, each signal channel contains an estimated target echo and corresponding interference components. Target detection is then performed independently on each channel using a Constant False Alarm Rate (CFAR) algorithm. In high-interference environments, however, detection results may exhibit significant deviations across channels due to varying local SNR conditions. To mitigate the potential accumulation of detection errors when aggregating across channels, the range measurements of each detected target are averaged over all signal channels to yield a more robust estimation of radial distance:

(28) $$D_{m}^{l}=\frac{1}{S}\sum_{i=1}^{S}d_{m,i}^{l}$$

where $D_{m}^{l}$ denotes the estimated radial distance from the *m*th PA radar to the *l*th target, and $d_{m,i}^{l}$ is the radial distance of the *l*th target as detected by the *i*th signal channel of the *m*th radar.

### 4.1. Cooperative Localization

Under low-SNR conditions, it is possible that only a subset of the distributed radar nodes detect the target, which necessitates adaptive localization strategies based on available detection information. Two cases are considered: localization using two radars, and localization using three or more radars.

Case 1: Localization Using Two PA Radars

Assume two PA radars located at $\left(x_{d1},y_{d1},z_{d1}\right)$ and $\left(x_{d2},y_{d2},z_{d2}\right)$, respectively, detect the target. Let the measured radial distances be $d_{1}$ and $d_{2}$, and the estimated DOA be $\theta_{1}$ and $\theta_{2}$. The target’s 3D coordinates $\left(x_{0},y_{0},z_{0}\right)$ can be obtained by solving the following system of nonlinear equations:

(29) $$\left\{\begin{array}{c} {\left(x_{d1}-x_{0}\right)}^{2}+{\left(y_{d1}-y_{0}\right)}^{2}+{\left(z_{d1}-z_{0}\right)}^{2}=d_{1}^{2}\\ {\left(x_{d2}-x_{0}\right)}^{2}+{\left(y_{d2}-y_{0}\right)}^{2}+{\left(z_{d2}-z_{0}\right)}^{2}=d_{2}^{2}\\ \theta_{1}=\arctan\left(\frac{y_{0}-y_{d1}}{x_{0}-x_{d1}}\right)\\ \theta_{2}=\arctan\left(\frac{y_{0}-y_{d2}}{x_{0}-x_{d2}}\right) \end{array}\right.$$

This system can be solved using iterative methods such as the Gauss–Newton algorithm to estimate the target position $\left(x_{0},y_{0},z_{0}\right)$.

Case 2: Localization Using Three PA Radars

Assume that three PA radars located at $\left(x_{d1},y_{d1},z_{d1}\right)$, $\left(x_{d2},y_{d2},z_{d2}\right)$ and $\left(x_{d3},y_{d3},z_{d3}\right)$ detect the same target, and the corresponding radial distances are $d_{1}$,$d_{2}$ and $d_{3}$, respectively. The spatial coordinates of the target $\left(x_{0},y_{0},z_{0}\right)$ can then be estimated by solving the following trilateration equations:

(30) $$\left\{\begin{array}{c} {\left(x_{d1}-x_{0}\right)}^{2}+{\left(y_{d1}-y_{0}\right)}^{2}+{\left(z_{d1}-z_{0}\right)}^{2}=d_{1}^{2}\\ {\left(x_{d2}-x_{0}\right)}^{2}+{\left(y_{d2}-y_{0}\right)}^{2}+{\left(z_{d2}-z_{0}\right)}^{2}=d_{2}^{2}\\ {\left(x_{d3}-x_{0}\right)}^{2}+{\left(y_{d3}-y_{0}\right)}^{2}+{\left(z_{d3}-z_{0}\right)}^{2}=d_{3}^{2} \end{array}\right.$$

Solving this overdetermined nonlinear system yields the 3D coordinates of the target, which can be efficiently computed using least-squares or iterative methods.

### 4.2. Downgrade Strategy

Collaborative ranging requires at least two independent radial measurements or two or more independent arrival direction measurements to obtain a three-dimensional position estimate. If fewer than two valid radial measurements are available, SDCAL employs a hierarchical fallback strategy: (i) attempt triangulation based on arrival directions when sufficient arrival directions exist, though with reduced reliability and (ii) return a “no positioning” state if a reliable geometric solution cannot be obtained.

This collaborative localization approach enhances the robustness and accuracy of target position estimation, particularly in scenarios involving partial detection or measurement errors among individual radar nodes. The complete algorithm flow is summarized in Algorithm 1.
**Algorithm 1:** Signal–data cooperative antijamming and localization (SDCAL)
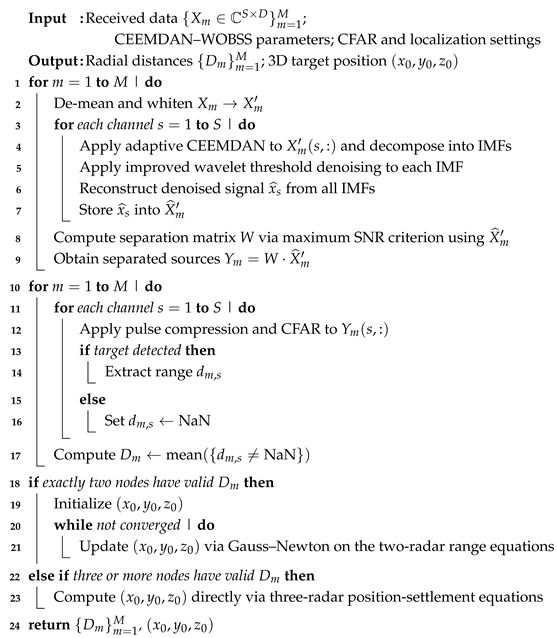


It should be emphasized that the SDCAL framework presented here integrates per-unit CEEMDAN decomposition and wavelet denoising, local multi-channel whitening followed by MSNR-BSS, pulse compression with CFAR detection, and centralized range fusion. The dominant computational burden arises from the local covariance and eigenvalue computations performed during CEEMDAN preprocessing and BSS. In practical deployments these operations are highly parallelizable across array elements and nodes, and the BSS at each PA node operates in a low-dimensional space. Therefore, with appropriate parameter tuning and hardware acceleration, real-time or near-real-time operation is attainable.

## 5. Simulation Results

The CEEMDAN–WOBSS + multi-channel BSS processing pipeline proposed in this work is effective provided the targets to be resolved lie within the physical resolvability limits imposed by the radar waveform and array geometry. Two primary, physics-driven resolution constraints should be noted.

First, the range resolution is governed by the transmitted bandwidth *B* and can be approximated by

(31) $$\Delta R\approx \frac{c}{2B},$$

where *c* is the speed of light. When the radial separation between two targets is substantially smaller than ΔR, their post-pulse-compression echoes overlap in range, and time-domain separation becomes intrinsically difficult. Second, the angle (DOA) resolution is constrained by the array effective aperture *L* (or equivalently by the number of elements *S* and inter-element spacing *d*), with a first-order estimate

(32) $$\Delta \theta \approx \frac{\lambda }{L}\approx \frac{\lambda }{Sd},$$

where λ is the carrier wavelength. Targets whose angular separation is significantly below Δθ will be difficult to distinguish by array-based spatial processing alone.

In addition to these geometric and waveform limits, source coherence degrades separability: highly coherent targets reduce the effectiveness of BSS and subspace methods. CEEMDAN preprocessing improves the effective SNR and helps to restore the rank structure of the signal covariance, but it cannot overcome the fundamental physical limits above.

All numerical experiments reported in this manuscript were conducted within the above resolvability constraints.

To evaluate the performance of the proposed method, a simulation scenario was constructed involving a distributed radar network composed of three PA radars and a single target of interest. The spatial coordinates of the radar nodes and the target are provided in Table 1. Each PA radar is equipped with six receiving elements, uniformly spaced at half-wavelength intervals. The transmitted signal is a linear frequency-modulated (LFM) waveform, expressed as:

(33) $$s\left(t\right)=\exp\left\{j2\pi \left(f_{c}t+\frac{1}{2}ut^{2}\right)\right\}$$

where $f_{c}$ is the carrier frequency and *u* is the modulation rate. The signal parameters are detailed in Table 2. The directions of arrival (DOAs) of the target and the interference are set to $10^{{}^{\circ}}$ and $11^{{}^{\circ}}$.

This paper defines two types of main lobe interference: NAM interference and SMSP interference. The NAM interference signal model is as follows:

(34) $$J_{\mathrm{NAM}}=\left(U_{0}+U_{n}\left(t\right)\right)\exp\left\{j(2\pi f_{j}t+\varphi )\right\}$$

where $U_{0}$ denotes the carrier amplitude, $U_{n}\left(t\right)$ is a zero-mean noise process with variance $\sigma^{2}=1$, ϕ is the initial phase, and $f_{j}$ is the carrier frequency of the NAM signal, set to 16 GHz.

The SMSP interference is modeled as a sequence of time-shifted subpulses:

(35) $$J_{\mathrm{SMSP}}=\sum_{i=0}^{I}J_{i}\left(t-\frac{iT_{p}}{I}\right)$$

where *I* denotes the number of subpulses and $T_{p}$ is the radar pulse duration. Each subpulse $J_{i}\left(t\right)$ is a short linear FM (LFM) burst defined on the interval $0\leq t\leq \frac{T_{p}}{I}$.

(36) $$J_{i}\left(t\right)=\exp\left\{j2\pi \left(f_{j}t+\frac{1}{2}\mu^{\prime }t^{2}\right)\right\},\;0\leq t\leq \frac{T_{p}}{I},$$

where $\mu^{\prime }=I\mu$ and μ is the linear modulation rate of the radar transmit waveform. Thus, each SMSP subpulse has the same instantaneous bandwidth as the radar LFM waveform, but a pulse duration reduced by a factor of $\frac{1}{I}$ and an effective chirp rate increased by a factor of *I*. In the simulations, the SMSP carrier frequency is set to $f_{j}=16\;\mathrm{GHz}$ and the number of subpulses is I=5. The SNR is set to 5 dB, the JNR is 50 dB, and the number of fast-time snapshots is 4096.

**Figure 3:**
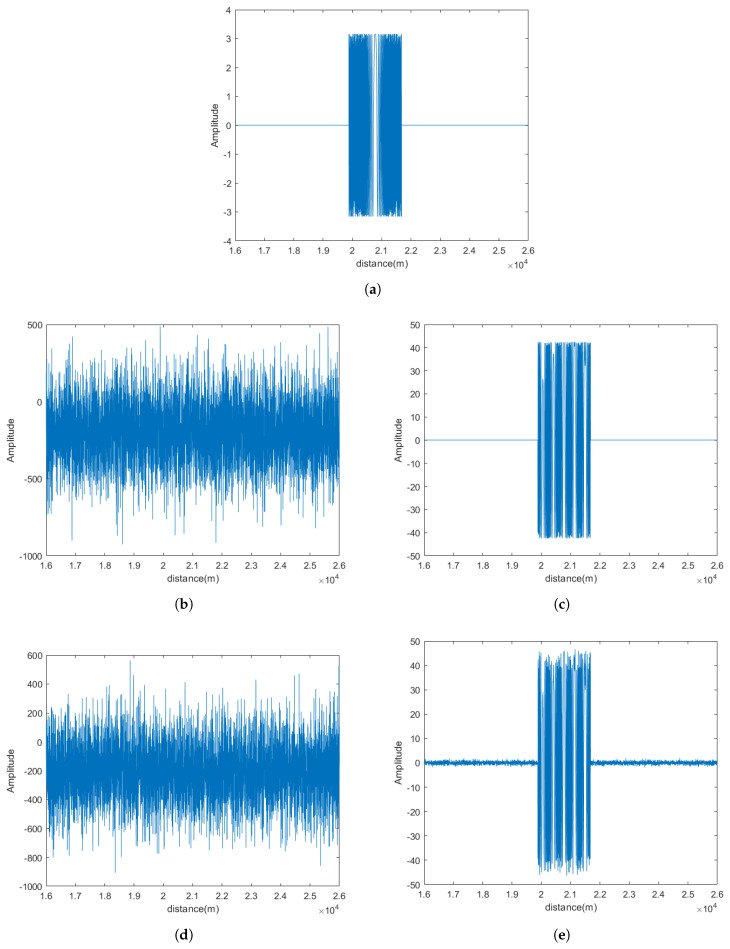
PA radar simulation signal model: (**a**) target echo signal; (**b**) NAM jamming; (**c**) SMSP jamming; (**d**) radar-received signal (NAM); (**e**) radar-received signal (SMSP).

To demonstrate the signal separation performance, Radar 1 is used as an example. In the proposed CEEMDAN-WOBSS method, CEEMDAN decomposition is performed up to 10 layers, and a six-level decomposition is performed using the improved wavelet basis function sym8. The target echo, NAM interference, SMSP interference, and radar-received signals are shown in Figure 3.

**Figure 4:**
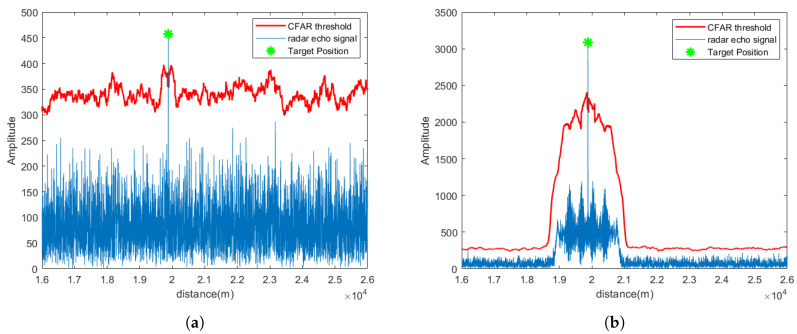
After the signal received by a single element of Radar 1 is separated by CEEMDAN-WOBSS, the pulse pressure and CFAR detection results are obtained: (**a**) under NAM interference conditions; (**b**) under SMSP interference conditions.

**Figure 5:**
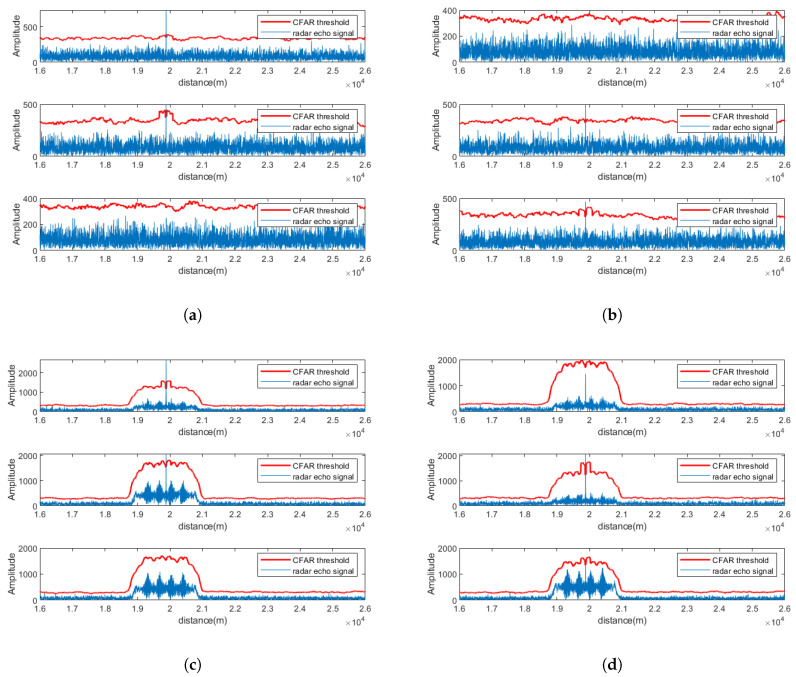
After the received signals from each array element channel of the PA radar are separated by CEEMDAN-WOBSS, the pulse compression and CFAR detection results are as follows: (**a**,**b**) Additional checks performed: results for channels 1–6 under NAM interference conditions; (**c**,**d**) results for channels 1–6 under SMP interference conditions.

Figure 4 depicts the pulse compression and CFAR detection results for a single channel after signal separation, while Figure 5 illustrates the results across all receiving channels of Radar 1. As seen from the figures, under SNR = 5 dB, the proposed method effectively suppresses strong electromagnetic interference and successfully separates target signals in multiple channels.

**Figure 6:**
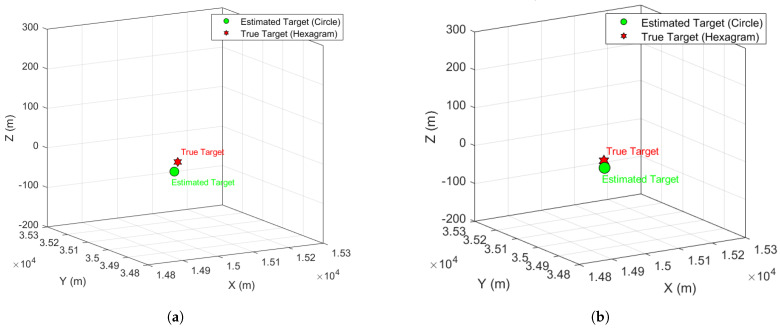
Spatial positioning results: (**a**) under NAM interference conditions; (**b**) under SMSP interference conditions.

After applying the CEEMDAN-WOBSS algorithm and CFAR detection to all three PA radars in the distributed radar network, the estimated radial distances of the target under both interference scenarios are listed in Table 3, with a maximum range error of only 2.5 m. Using the position estimation formulas discussed earlier, the 3D target location is calculated, as shown in Figure 6.

In practical radar detection environments, the received SNR often ranges from −5 dB to 5 dB. Thus, this paper compares the interference suppression performance of the proposed CEEMDAN-WOBSS method against MSNR-BSS under JSR=50dB and SNR∈[−5dB,5dB]. The comparison includes evaluations of the peak side-lobe ratio (PSLR), target detection probability, and localization accuracy across various SNR levels. Additionally, the JADE-BSS method and MSNR-BSS are used as a baseline for benchmarking.

### 5.1. Signal Separation Performance at SNR = −5 dB

**Figure 7:**
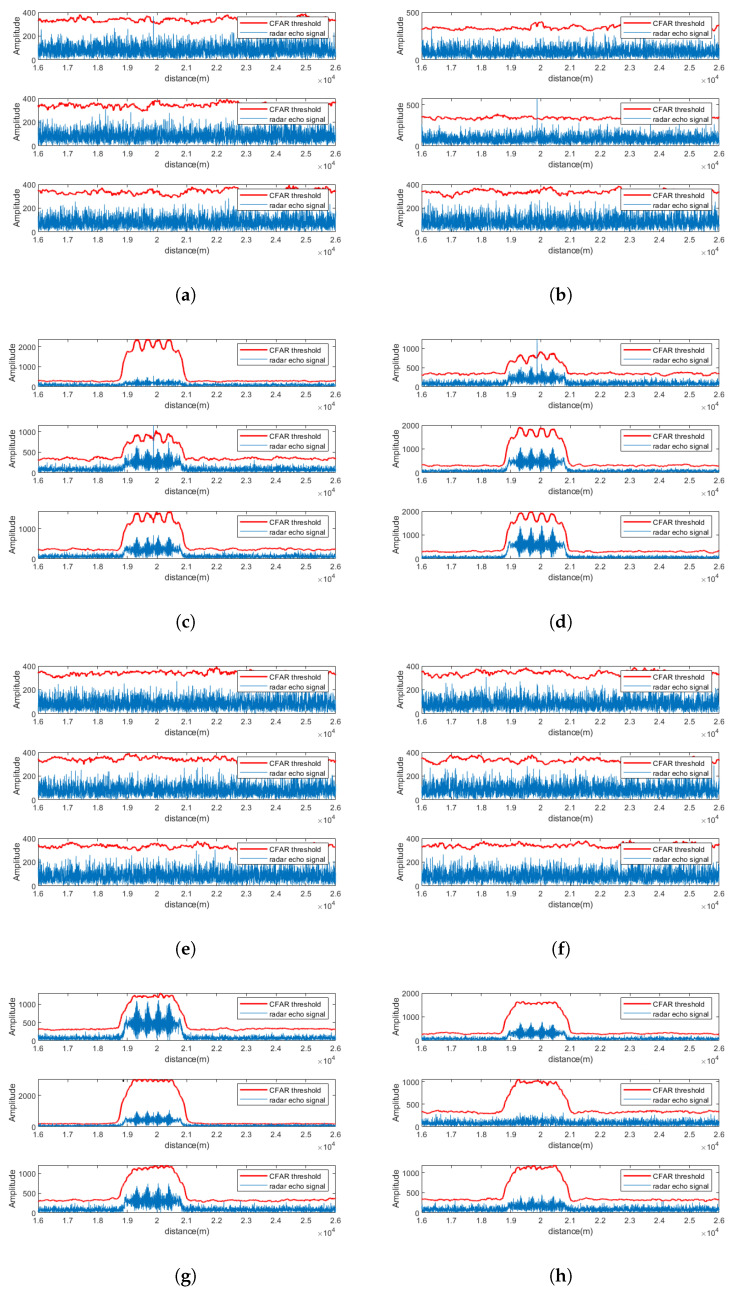
SNR = −5 dB. PA radar 1 received a signal after separation by the CEEMDAN-WOBSS method and MSNR-BSS method; the pulse compression and CFAR detection results are presented for the following: (**a**,**b**) Additional checks performed: CEEMDAN-WOBSS method, channels 1–6 (NAM); (**c**,**d**) CEEMDAN-WOBSS method, channels 1–6 (SMSP); (**e**,**f**) MSNR-BSS method, channels 1–6 (NAM); (**g**,**h**) MSNR-BSS method, channels 1–6 (SMSP).

Using Radar 1 as an example, the pulse compression and CFAR detection results of CEEMDAN-WOBSS under two interference are shown in Figure 7a–d, respectively. For comparison, the results of the MSNR-BSS method are shown in Figure 7e,f. The results indicate that under low-SNR conditions (−5 dB), MSNR-BSS fails to recover the target echo in any channel, whereas CEEMDAN-WOBSS successfully separates and detects the target signal in several channels, effectively suppressing the NAM interference.

### 5.2. Signal Separation Performance at SNR = 0 dB

**Figure 8:**
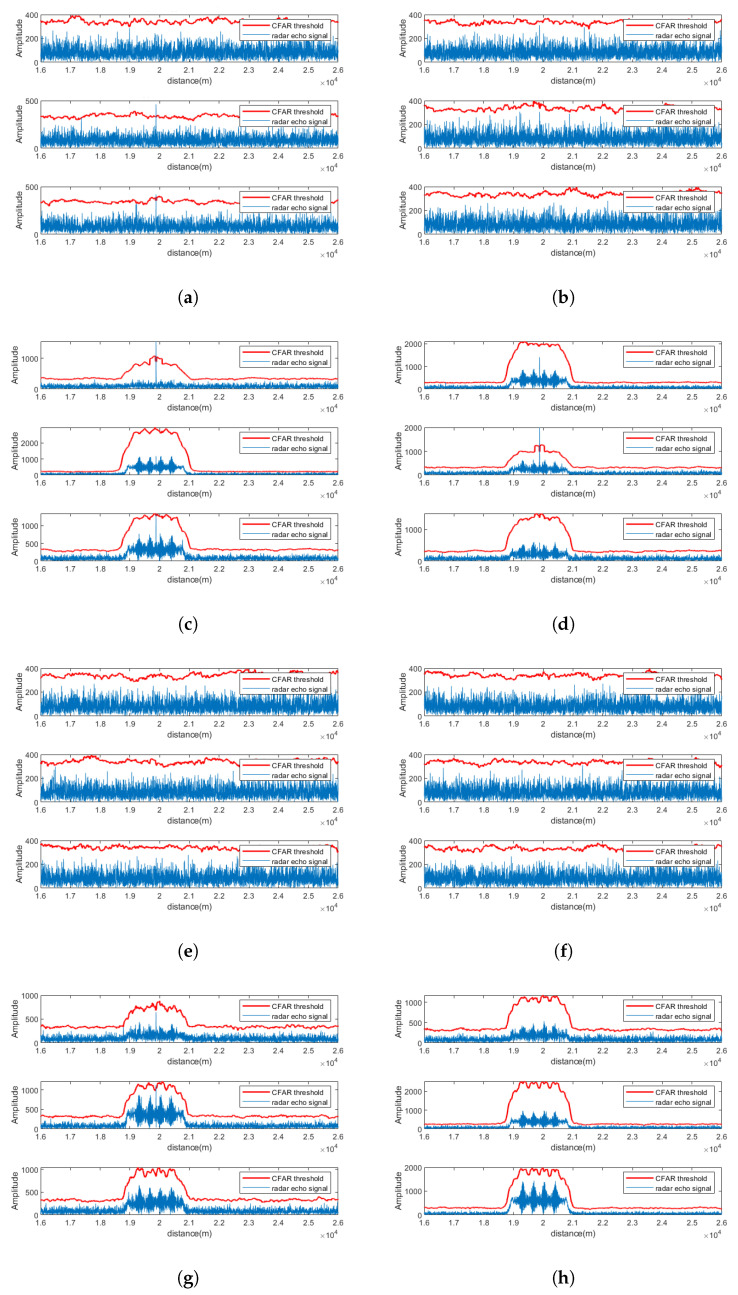
SNR = 0 dB. PA radar 1 received a signal after separation by the CEEMDAN-WOBSS method and MSNR-BSS method; the pulse compression and CFAR detection results are presented for the following: (**a**,**b**) Additional checks performed: CEEMDAN-WOBSS method, channels 1–6 (NAM); (**c**,**d**) CEEMDAN-WOBSS method, channels 1–6 (SMSP); (**e**,**f**) MSNR-BSS method, channels 1–6 (NAM); (**g**,**h**) MSNR-BSS method, channels 1–6 (SMSP).

Figure 8 presents the pulse compression and CFAR results for the CEEMDAN-WOBSS method under two interference scenarios at 0 dB SNR. The corresponding results for MSNR-BSS are shown in Figure 8e,f. The simulation results demonstrate that CEEMDAN-WOBSS continues to effectively isolate and detect the target echo even at an SNR = 0 dB.

### 5.3. Signal Separation Performance at SNR = 5 dB

**Figure 9:**
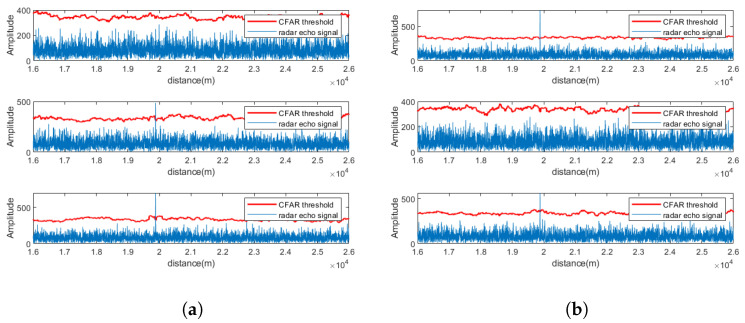

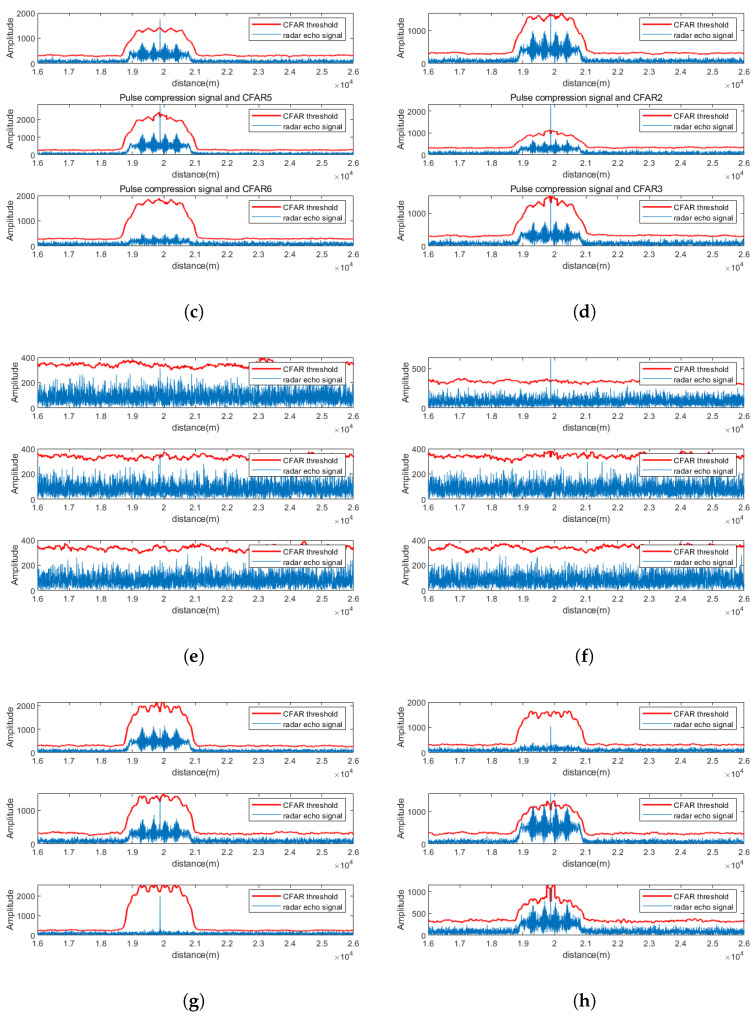
SNR = 5 dB. PA radar 1 received a signal after separation by the CEEMDAN-WOBSS method and MSNR-BSS method; the pulse compression and CFAR detection results are presented for the following: (**a**,**b**) Additional checks performed: CEEMDAN-WOBSS method, channels 1–6 (NAM); (**c**,**d**) CEEMDAN-WOBSS method, channels 1–6 (SMSP); (**e**,**f**) MSNR-BSS method, channels 1–6 (NAM); (**g**,**h**) MSNR-BSS method, channels 1–6 (SMSP).

Figure 9 show the pulse compression and CFAR detection results of CEEMDAN-WOBSS and MSNR-BSS under two interference scenarios at a 5 dB SNR. At this level, both methods can detect the target echo in multiple channels; however, CEEMDAN-WOBSS outperforms MSNR-BSS by recovering the target signal in more channels and providing better interference suppression.

### 5.4. Quantitative Evaluation of Effectiveness

To quantify the performance of the CEEMDAN-WOBSS method, three metrics were adopted under the setting of JSR=50dB and SNR∈[−5dB,5dB]:

1. Peak side-lobe ratio (PSLR): The PSLR is defined as
  (37) $$\mathrm{PSLR}=20\log_{10}\left(\frac{\mathrm{SLP}}{\mathrm{MLP}}\right)$$
  where SLP is the peak amplitude of the side lobe and MLP is that of the main lobe. The average PSLR across *N* Monte Carlo trials is given by
  (38) $${\mathrm{PSLR}}_{N}=E\left({\mathrm{PSLR}}_{n}\right)$$
  where E(·) denotes the expectation operator.
2. Target Detection Probability, defined as
  (39) $$P_{d}=\frac{N_{\mathrm{detect}}}{N_{\mathrm{total}}}$$
  where $N_{\mathrm{detect}}$ is the number of successful detections and $N_{\mathrm{total}}$ is the total number of Monte Carlo simulations.
3. Localization Accuracy, measured by the 3D Euclidean distance between the estimated and true target positions:
  (40) $$E_{3D}=\sqrt{{\left(x_{\mathrm{est}}-x_{\mathrm{true}}\right)}^{2}+{\left(y_{\mathrm{est}}-y_{\mathrm{true}}\right)}^{2}+{\left(z_{\mathrm{est}}-z_{\mathrm{true}}\right)}^{2}}$$
  The average localization error over *N* simulations is then calculated as
  (41) $$E_{3D}^{N}=E\left(E_{3D}^{n}\right)$$

**Figure 10:**
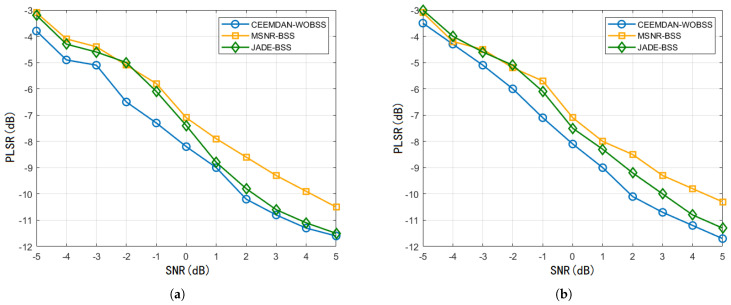
JSR=50dB and SNR∈[−5dB,5dB]. PSLR results of the three methods (**a**) under NAM interference conditions and (**b**) under SMSP interference conditions.

Under a fixed JSR=50dB and SNR∈[−5dB,5dB], 100 Monte Carlo trials were executed for each SNR value under two interference scenarios to compute the average PSLR. As illustrated in Figure 10, the average PSLR of the separated target signal produced by all three algorithms decreases monotonically with an increasing SNR, indicating that higher SNR levels yield improved separation performance. Notably, CEEMDAN–WOBSS achieves a PSLR reduction of 0.2645–0.6235 dB compared to MSNR-BSS and JADE-BSS.

**Figure 11:**
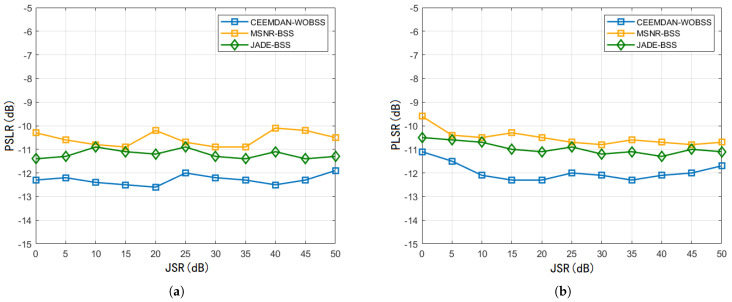
SNR=5dB and JSR∈[0dB,50dB]. PSLR results of the three methods (**a**) under NAM interference conditions and (**b**) under SMSP interference conditions.

Figure 11 shows the average PSLR under two interference scenarios at textSNR=5dB for JSR∈[0dB,50dB]. The minimal variation in PSLR across this JSR range demonstrates that moderate changes in interference power do not significantly affect the separation performance of CEEMDAN–WOBSS.

**Figure 12:**
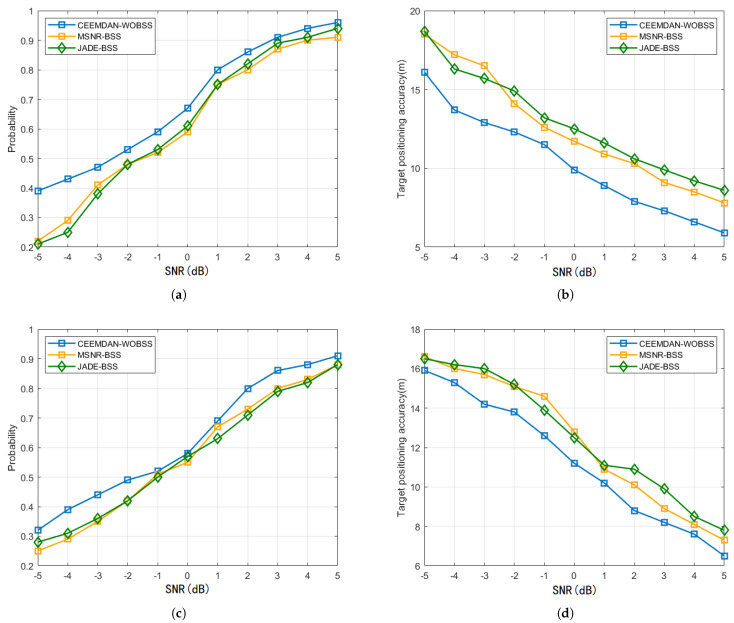
JSR=50dB and SNR∈[−5dB,5dB]. Results of the three methods for detecting probability and positioning accuracy: (**a**,**b**) detection probability and localization error in NAM interference scenarios; (**c**,**d**) detection probability and localization error in SMSP interference scenarios.

Using the same JSR=50dB and SNR∈[−5dB,5dB] conditions, Figure 12 compares the target detection probabilities and cooperative localization errors of the three methods across two interference scenarios. The proposed CEEMDAN–WOBSS method improves the detection probability by 2–17% and enhances cooperative localization accuracy by 8.1–27.6% relative to MSNR-BSS and JADE-BSS.

**Figure 13:**
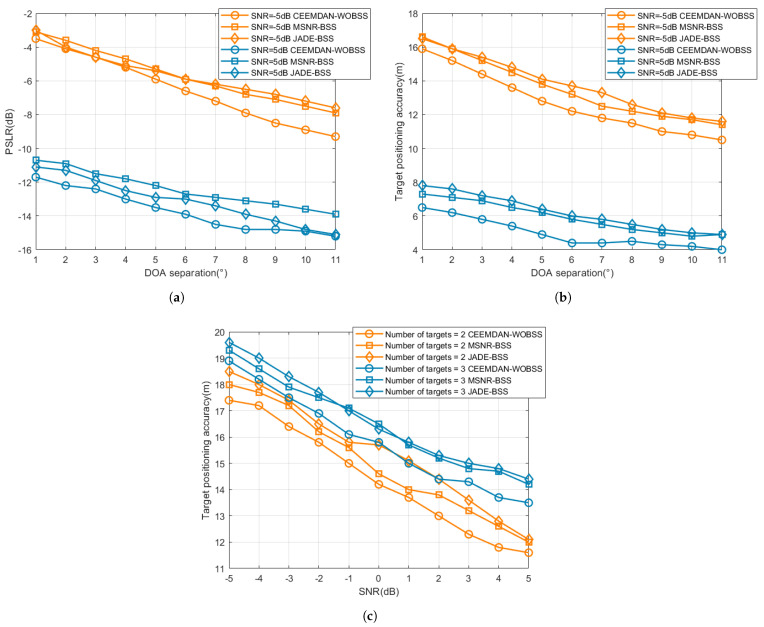
Interference-suppression and localization performance under varying angular separation and multi-target conditions. The angular separation between each target DOA and the jammer DOA was varied from $1^{\circ }$ to $11^{\circ }$, and rgw SNR was varied from −5 dB to +5 dB. Results are averaged over 100 Monte Carlo realizations: (**a**,**b**) SNR=−5dB and 5dB; variation in PLSR and positioning accuracy with angle of arrival difference for the three methods; (**c**) SNR∈[−5dB,5dB], average positioning accuracy of the three methods under two-target and three-target scenarios.

To assess the proposed method’s interference-suppression and localization performance under varying angular separations and multi-target conditions, we computed and statistically analyzed the PSLR and localization error as the angular separation between each target DOA, varying the jammer DOA from $1^{\circ }$ to $10^{\circ }$. Additionally, interference-suppression and localization tests were performed for two- and three-target scenarios in which the targets shared the same DOA but were separated by 1000 m in range. In each Monte Carlo trial we recorded the mean localization error across all targets; the reported statistics were aggregated over 100 Monte Carlo realizations. The interference type was NAM interference, with all other experimental parameters remaining as specified above. The resulting performance curves are presented in Figure 13.

The results in Figure 13 indicate that as the arrival angle separation increases, the PSLR value rises while the positioning error decreases; moreover, a higher signal-to-noise ratio simultaneously improves both metrics. Under all conditions, the proposed CEEMDAN–WOBSS method achieves the highest PSLR value and lowest positioning error, with all three methods converging under conditions of large separation and high SNR. In multi-target tests, all methods exhibited performance degradation, with errors gradually decreasing as the SNR increased. CEEMDAN–WOBSS demonstrated a larger reduction in error, indicating superior robustness in dense target scenarios.

Furthermore, to demonstrate that the proposed method achieves a superior antijamming performance and localization accuracy under low-SNR conditions, a statistical comparison of positioning errors was carried out for both the proposed SDCAL pipeline and a single-station radar across a range of SNR values. For the single-station baseline, the target DOA was first estimated using a standard DOA estimation algorithm prior to localization. The numerical results are summarized in Table 4.

As shown in Table 4, the proposed framework effectively enhances the spatial localization accuracy of targets under low-signal-to-noise ratio conditions. Compared to relying on a single radar for target detection and localization, the proposed SDCAL framework enables data-level collaborative localization. This approach can tolerate partial node detection failures while still achieving localization based on two or more reliable nodes.

## 6. Discussion

In this paper, an SDCAL framework for mainlobe interference suppression and three-dimensional target localization in distributed radar networks is proposed. To address the challenges posed by low-SNR environments, the CEEMDAN–WOBSS algorithm is first employed at each PA radar node to decompose, denoise, and reconstruct the received echoes, thereby reducing the noise baseline and restoring the effective rank of the signal covariance matrix. A maximum-SNR-based separation matrix subsequently extracts the target echo, which is then refined through pulse compression and CFAR detection to suppress residual interference. Finally, the fusion of range measurements across multiple nodes yields precise spatial coordinates. The simulation results show that SDCALF markedly enhances interference suppression, leading to significant gains in target detection probability and localization precision under low-SNR conditions when compared with conventional BSS schemes. Moreover, even in the presence of typical suppression jamming (such as noise frequency modulation and noise phase modulation), the proposed method maintains robustness and stable localization performance.

This study primarily serves as a proof of concept to validate the core signal processing chain under challenging yet specific scenarios. It should be noted that this research has certain limitations: the current implementation assumes a known node geometry and standard clock synchronization; significant synchronization errors or uncalibrated geometries will degrade performance unless compensated for. Source counting processing occurs outside the main processing pipeline, and misclassifications may cause separation and localization failures. The current fusion rules employ simple averaging/aggregation algorithms and have not been optimized for damaged or adversarial node reports. Regarding computational resource consumption, the CEEMDAN and BSS phases are the most resource-intensive, requiring algorithmic and implementation optimizations for real-time deployment. Finally, experiments were conducted only on limited static scenarios and jammer types; performance may differ in dense target scenarios near array resolution limits, for high-maneuver targets, or in complex multipath/clutter environments. We have prioritized addressing these issues in future work and will focus on enhancing the method’s operational robustness and deployability.

## Figures and Tables

**Table 1:** PA radar spatial coordinates.

Radar Serial Number	Position Coordinates (m)
PA Radar 1	8000, 22,000, 15,000
PA Radar 2	10,000, 20,000, 15,000
PA Radar 3	8000, 24,000, 15,000
Target location	15,000, 35,000, 0
Interference location	15,500, 35,600, 0

**Table 2:** Radar parameter settings.

Signal Parameter	Parameter Value
Bandwidth/B	20 MHz
Carrier Frequency/Fc	16 GHz
Pulse Repetition Frequency/PRF	8 MHz
Sampling Frequency/Fs	80 MHz
Number of Sampling Points	4096
Pulse Width/Tp	25 μs
CEEMDAN decomposition level	10
Wavelet basis	sym8
Wavelet decomposition level	6
balancing parameter α	2
CFAR type	CA-CFAR
CFAR false alarm probability	$10^{-5}$
Number of reference units	50
Number of protection units	5

**Table 3:** Distributed radar network node radar positioning distance.

Radar Number	Actual Distance (m)	Estimated Distance (NAM) (m)	Estimated Distance (SMSP) (m)
PA Radar 1	21,048	21,050.1	21,049.9
PA Radar 2	21,794	21,796.4	21,796.1
PA Radar 3	19,875	19,877.5	198,877.4

**Table 4:** Positioning error of single-site radar and multi-site radar.

Positioning Method	SNR = −5 dB (m)	SNR = 0 dB (m)	SNR = 5 dB (m)
Single-site radar	18.6	12.3	7.6
Multi-site radar	16.1	9.8	5.9

## Data Availability

Data are contained within the article.
